# The Importance of Blood Pressure in Clinical Medicine

**Published:** 1905-10-14

**Authors:** T. Clifford Allbutt

**Affiliations:** Regius Professor of Physic at the University of Cambridge


### Hospital Clinics.

### THE IMPORTANCE OF BLOOD PRESSURE IN CLINICAL MEDICINE.

By T. Clifford Allbutt, M.D., F.R.S., Regius Professor of Physic at the University of Cambridge.

When I invented the pocket clinical thermometer, and so put into the hands of every physician what had been till then a curious implement, rather like a small umbrella, found in Great Britain only in some three or four hospitals, it was for some time contemptuously neglected by practical men as a philosophical fad. He, they said, who could not teli when a patient was feverish without arming himself with this whimsical thing was devoid of clinical perception, and of the *tactus eruditus*. Those objectors have gone down the stream of time. But their kind is not extinct; now we are assured that he who cannot with his finger tell arterial pressures is a dunce. These obscurantists, capable men as many of them are, in spite of their deafness to the claims of advancing science, will likewise go down the stream, and their protests will be forgotten, or quoted by the historian only to point a moral. Of the two conditions I am disposed to think that fever and degrees of fever are easier to ascertain with the unaided hand than those of arterial pressure. Almost as I write a physician of considerable skill and experience, and one awake to the importance of arterial pressure, brought a patient to me, saying in our preliminary discussion that, although in this case he had anticipated a rise of pressure, yet in his opinion —judging by the finger—the pressure was not raised. And on my own examination I, too, was far from sure of the contrary, but as the pulse was sustained too long I suspected my friend to be wrong; and on verification the systolic pressure amounted to no less than 210 mmHg. Now, in the present state of our instruments, I admit that we may easily exaggerate our means of registering arterial pressures to degrees of close accuracy; for my own part I scarcely claim more than to say whether such pressure be subnormal, normal, moderately enhanced, much, very much, or, in some extreme cases, enormously enhanced. I do not enter estimates in my notes by millimetres, or do so only as approximate values. Still, in clinical diagnosis, as we shall see, even approximations are to be regarded as of prime importance. As an example of the deceptiveness of the finger for even substantial differences of pressure, I often ask my confident friends to touch first the radial artery and then the brachial, and to compare the pressures; if they are frank they admit at once that that of the brachial seems to the finger by far the higher, yet I need not say that the difference is really inappreciable. On the other hand, such is the imposition of diameter on the finger touch, a much contracted radial conveys less sense of pressure, *cæteris paribus*, than a distended vessel; in the contracted vessel a very high pressure may escape digital appreciation. Thus the well-known large pulse of the elderly gives a sense of tension often far beyond the truth. Again, we are told, that the loudness and quality of the second aortic sound and the size of the heart are sufficient guides to arterial pressure; this is not so: a loud and far-carrying second aortic sound is heard in many healthy persons, especially in women; a " tabourka " quality of the second sound signifies atheromatous parts, whatever the pressure. Incipient degrees of hypertrophy of the heart are most difficult of appreciation, and it is in the incipient stages that we must detect rising pressures if we are to prevent them and their consequences.

As to instruments it would take us in this short essay too far to compare them in detail; I can give but a few personal suggestions. An aneroid dial has less inertia, is easier to read and to carry about than a mercurial column; on the other hand it is said to be less trustworthy. For close laboratory work a mercurial column is, of course, preferable; but for the approximate registers of ordinary clinical observation I find the aneroid of Hill and Barnard's instrument keeps true enough if verified by the maker two or three times a year—no difficult matter. The two serious drawbacks to Hill and Barnard's instruments are: (1) that the armcuff is too narrow, and to my instrument I have adapted a cuff of five inches width; (2) that for the sort of bicycle pump supplied with it two hands are required, so that it is not easy to keep a finger free for the radial artery—an essential condition; a ball-pump, which can be worked with one hand, is, therefore, much better. Of the mercurial or Riva-Rocci instruments, Janeway of New York has produced a convenient one, but the ball is unnecessarily large, and the box frame is of such bad workmanship as to make the use of it a perpetual fidget. If Dr. George Oliver's new instrument prove efficient it will be a very convenient apparatus.

The feature of pressure which chiefly we desire to ascertain in practice is the systolic pressure; the relation of the pause to systole and diastole, the sustenance of the pulse wave, and the diastolic pressure are not well indicated by the sphygmometer. An undue duration of the wave, an important feature, is generally perceptible to the finger; but in a difficult diagnosis I take a sphygmographic tracing also, being careful not to compress the artery beyond the degree at which the amplest tracing is obtained. Thus the relations of systole to diastole and pause can be compared, and, indeed, some notion of the state of the arterial wall obtained also.

The patient may be seated comfortably at a table, the arm resting easily on it at the level of the heart. His nerves must be as tranquil as his muscles. As the means and object of the instrument are explained to him his attention is diverted from himself. There is no objection to the soft underclothing on a woman's arm; and the need of the instrument is, in my experience, as frequent in women as in men. In children the method can be but occasional; in little children it is taken from the thigh.

The pump is slowly urged until the pulse disappears at the wrist; and although by percussion on the upper rim of the cuff the needle or column may still oscillate, this is the systolic pressure. It is best to stop the artery two or three times, noting on each occasion the figure at which it returns to the finger.

Into the daily or occasional variations of arterial pressures in the healthy I cannot enter; under the management I have prescribed normal fluctuations will create no difficulty. However, there is a secular change of great practical importance: namely, that in the advance to middle age and after pressures slowly rise. The systolic pressure which in an active undergraduate may be at or even under 100 mm., will, as age advances, increase to 120, say, at thirty, 130 at forty, 150 at sixty. Such stresses are in respect of their ages not to be regarded as more abnormal than the mortality of mankind. But a pressure of 140 in a man of thirty is very suspicious, 150 certainly morbid. In elderly men pressures of 160 and upwards point to some disorder, and must be closely watched. In disease, at any age, as in Bright's disease, pressures may rise very often to 200 and over; in Hill and Barnard's instrument I never press the pump beyond 230, for fear of straining my aneroid, but by the mercurial column I frequently read systolic pressures of 250. In cerebral compression, tending to express the blood from the bulb, the heart may drive the compensating pressures up to 400 and more (Cushing). Arterio sclerosis affects the pressure only to some 5 mm., and may be neglected.

It is to Professor von Basch that we owe the first extensive work on arterial pressure in clinical medicine; to him our debt is very great. But fifteen years ago I found myself unable to accept his interpretation of the relation of arterio-sclerosis to high pressures. The rise of pressure in middle and elderly persons was attributed by this eminent observer, and all other writers have followed him, to arterial disease. If the pressures rose in persons in whom no such disease was discoverable—well, there was "latent arterio-sclerosis." Now I found that rise of pressure to heights definitely morbid was no very uncommon event in young persons, even in children, in whom arterial disease was usually out of the question. Again, after watching many cases over such periods as twelve to eighteen years, I found with the lapse of time that rise of arterial pressure was followed, after the lapse of some years, by arterio-sclerosis, so that this static condition, if perchance it might be the extension into our ken of arterio-sclerosis pre-existing elsewhere, was more likely to be the effect of prolonged abnormal stress upon vulnerable vessels—whether vulnerable, as all tissues must be, or particularly vulnerable in certain individuals. Furthermore I found, and demonstrated continually to my classes, both in Leeds and in Cambridge, the presence of arterio-sclerosis unattended by any rise of pressure, as in young persons afflicted with syphilis or diabetes, for example; or in elderly persons in whom some increase did not exceed that usual in healthy persons of advancing years. In this opinion I found not a single disciple until the last year or two; by all physicians it was urged that, when the arterial pressure rose in persons in whom the accessible arteries were fairly healthy, it was because of arterio-sclerosis in the vessels of within, mainly in, the mesenteric area, whereby the regulating capacity of this area was abated. Against this argument I was able to urge that this area becomes the seat of atheroma very early in the course of such degenerations in all elderly persons, whether their pressures be raised or not; and that my pupils had collected large numbers of cases of old persons in whom arterio-sclerosis in this abdominal area was well advanced, and yet without any significant rise of pressure. Now I think the opinion of pathologists is wavering, and beginning to admit that arterio-sclerosis and rise of blood pressure do not stand necessarily in the position of cause and effect; but, as arterial pressure may rise long before any deterioration of the arterial walls, so the arterial walls may be universally diseased, including the mesenteric area, without more than that moderate rise of pressure which is characteristic of all older life.*

If then, rise of blood pressure and arterio-sclerosis may occur independently of each other, what are the causes of each of these changes, respectively, a dynamic and a static change? Before we touch this point I would say that, to the physician who is awake to the matter, a rise of arterial pressure is no unusual disorder. As I have said, it is no infrequent event in children, and in them, apart from some peculiar toxic factor, such as lead, passes away without permanent harm, seeming, as it does, to depend on some temporary derangement of the primæ viæ, which now we have not time to consider. Likewise it very often occurs in elderly persons, when, if care be taken to neutralise the causes of it, in them too it passes away, leaving no permanent harm. Such elderly patients may or may not be the subjects of obvious arterial degeneration; in either case an incidental hyperpiesis, as I have ventured to name it, alters the static conditions but little, or not at all. At the same time it is scarcely necessary to say that where the vessels are decaying or strained an access of hyperpiesis is perilous.

If, then, hyperpiesis is not the effect of preceding arterio-sclerosis, as is ordinarily taught, what is the cause of it? Here we are in a difficulty. We have, indeed, no definite notion; so far our criticism is but destructive. Or, again, if we ask what is the cause of arterio-sclerosis, the question is no less difficult. Now, as I have often put it, if the high pressure results from increased resistance at the periphery—an assumption which may be said to hold the field in respect of the cases we have under our consideration—it can be due to one cause only—namely, an increase in friction. Now an increase in friction may consist in an increase in friction within the blood itself—in an increase, that is, of its viscosity, or in a narrowng of the channels of the blood. There is some expert evidence of increased viscosity in Bright's disease with high pressure; but, speaking generally, such increase is far from being verified in

* *Cf.* a recent able article in the *Clinical Journal* by Dr. Rolleston, my note of the date of which is mislaid.

arterio-sclerosis. Is it, then, a narrowing of the channels of the arteries? If so, it must be over very large areas, a constriction practically universal, or the ordinary machinery for compensating constriction of the arteries in extensive areas—namely, the splanchnic depressor machinery—must be in abeyance, as perchance by some defect in nervous connection or conduction, or, again, by some inability of the vessels in this area to dilate on demand. Of the power of universal arterial contraction to raise pressures to widely abnormal degrees there is no doubt; of the defect of the depressor machinery we have no evidence. A glass of wine or a dose of a nitrite appears to be as effective in dilating vessels in an old man as in a young one. But it is not my intention to enter into this argument, only to indicate the denser parts of our ignorance.

Of the clinical experience, however, that in the presence of some distempers pressures do rise there is no doubt whatever; how they act is a matter to be ascertained hereafter as we may. The finger on the pulse gives a hint, or the patient complains of symptoms which to the instructed observer are suggestive; the sphygmometer is applied, and the fact is verified. Now if this effect be continuous over a long period, and there is no doubt it may so persist for years without driving the patient to the physician, its effect upon the vessels must be destructive; and, as a matter of daily experience, we find it so. The pressure tells on the coats of the vessels, distending them at first with the same effect of setting up a compensating hypertrophy, as in the homologous case of the heart, and afterwards also (as in the heart) leading to degenerative disease. The arteries are distended beyond their elastic limits, and strained.

Now it is a common prejudice that a diseased arterial system is in itself a direct means of shortening life; a means of diminishing the value of life it may be, but a visit to any workhouse will provide examples at will of old men and women in whom advanced arterial decay has proved consistent with survival to extreme old age. On the other hand, many die but little after middle life of the effects of vascular disease—of apoplexy, for instance, or of angina pectoris—in whom, however, the arterial decay was very far less general. Where lies the difference? In this: that in those the mean arterial pressures had been low; in these so high as either to institute arterial disease, or, having supervened upon a foregone decay, to try some vessel beyond its reduced power of resistance. The immediate factor of death, then, is not the arterial disease, but the high pressure. The high pressure may have been due to some transient cause, such as an intense emotion or a violent effort, or to some persistent internal distemper. At one time I had to prove that these fatalities do not all belong to Bright's disease; but this contention is now settled: there is a large class of these cases of hyperpiesis which is independent of Bright's disease.

In former papers I have undertaken to classify arterial disease under causation, thus: (1) the *arterial disease of involution*, not due to hyperpiesis, nor attended by it, save by chance recurrence; (2) *the arterial disease of hyper*[illegible] in which the change is due directly to the persistence of high blood pressure; (3) *the arte*[illegible] *disease of toxic origin*, as lead, syphilis, typho[illegible] fever, diabetes, and so forth, not due to high bloo[illegible] pressure nor usually attended by it. The first class offers the most grotesque distortions of the vessels (if I may use such a word), and a well marked tendency to calcification, as well of the middle coat as of the inner. In the third class the thickening of the vessels is to be detected rather by the finger than by the eye, at any rate in young people. The evidences of the second kind of fault occupy an intermediate position. It is a common experience to detect a rise of arterial pressure in an old person, whether with obvious arterio-sclerosis or not, who may complain of certain symptoms; and it is clearly of the utmost importance in such elders to recognise the complication and to remove it at once. Fortunately in them it is often far more easy to get rid of than in those younger persons in whom the proclivity is probably more pronounced. In none of us do such periods of disorder with hyperpiesis fail altogether to arise, though we may never be made aware of it, unless by some sudden catastrophe. In old persons the slighter disturbances of function are more carefully watched and brought under medical care. Often in such cases may the impending apoplexy, or angina pectoris, or cardiac strain, or pneumonia, be averted by the proper therapeutical means. In the children whose high arterial pressure is usually or always due to a perversion of the gastric or intestinal order, with "biliousness," no such catastrophe is, of course, to be apprehended; the condition is removable by deobstruent treatment and regulation of diet. These children have coated tongues, irregular and offensive stools, foul breath, and distended stomachs which splash or squelch when the epigastrium is prodded.

The symptoms of the hyperpiesis in the middle-aged which leads to permanent injury to the arterial tree are, unfortunately, indefinite, so indefinite that I urged at the Royal Institution that every person passing forty-five should have his arterial pressure taken, and even if found normal nevertheless taken again and again, at intervals of five years or sooner, if he should feel sluggish, nervous, depressed, or "bilious," to the end of his life.* If discovered in time the hyperpiesis is usually reducible without much difficulty, and subsequently easily prevented. By rule of thumb many men go regularly year by year to certain spas at home or abroad, such as Carlsbad or Homburg, to clean the works, as they put it; that is, to dissipate the crudities which embarrass the circulation, either by clogging it or by setting up a contraction of the arteries over very large areas, and to arouse the organs of conversion and excretion which should reduce and discharge such impurities. Every reader of von Basch's books will perceive how commonly these frequenters of spas are the subjects of enhanced arterial pressure—atheroma or no atheroma.

In them the heart hypertrophies for the additional work, but—as I pointed out in 1869 in respect

* In this paper I cannot enter into the symptoms.

[illegible]ain of the heart—a hypertrophied heart is not [illegible]ood as a normal one. A long persistence of ex-[illegible]sive pressures dams back the output of the left [illegible]entricle, distends it, and gradually strains it. It is not quite fair to this faithful organ to include it—as Monsieur Huchard does—in the decadence of which it is the victim. It is astonishing, indeed, how long an obviously failing heart may still keep up, more or less, supernormal pressures, lavishing its marvellous reserves even to the bitter ned.

In conclusion, what is to be done for these very common cases of rising arterial pressures so as not only to remove the primary condition itself, but also to prevent those consecutive static alterations which at no very advanced stage become irremediable. Too often when a patient submits himself to examination we find in the accessible arteries of the arms and legs evidences of the irremediable injury of strain. But we must not be too ready to assume that a thickened artery always means a permanent alteration. In the enhanced pressures of children, for instance, we may find the radial and other superficial arteries to be thickened; but, after a proper course of treatment, this thickening wholly disappears. I suggest that in these cases the thickening is not arteriosclerosis but that hypermyotrophy which has been demonstrated by many observers, such as George Johnson, and more recently by Dr. Savill. As hypertrophy of the heart disappears on removal of its causes, so we may suppose a disappearance of an arterial hypermyotrophy when excessive lateral pressures are reduced. In adults, of middle life or under, we may note likewise a solution of arterial thickening as excessive pressures are reduced. Unfortunately, the more frequent experience is far otherwise; when the patient's condition is recognised, the arteries have undergone a permanent deterioration—that subepithelial degradation, or process of backsliding repair—which is of like nature to the atheroma of the ascending aorta and here confidently attributed to mechanical causes acting upon a wall perhaps more or less infirm already. Ignorant as we are of some of the principal conditions of the problem, we may well understand, as experience tells us, that with an arterial tree permanently altered the patient may be beyond complete repair, however successful we may be in arresting or retarding the course of the mischief. But henceforth neither in structure nor in function can the circulation become normal again. Immediate peril may be indefinitely postponed, but hardly so far as to permit the extension of life on what we may call the original scheme.

Unfortunately, many persons seem to have a tendency to excessive arterial pressures implicit in this original scheme, for fork these away as we may —*tamen usque recurrunt.* Few persons, as we have said, escape some transient phases of hyperpiesis; the causes are temporary, and the distemper passes, as it were, in the night; in them Nature has taken care of herself. But when elderly persons, whose machinery may no longer be of the soundest, are still subject to such tides of high pressure, and possibly they are more so, we cannot contemplate intercurrent stresses with a like equanimity. Transient as the rise may be, it may last just long enough to strain the heart, break a vessel of the brain, or invite a pneumonia. Happily, we note in practice that these phases are as easy to remove in the aged as in the middle-aged; perhaps easier in so far as survival to advanced years with fairly sound parts has depended on no great proclivity to err in the direction of hyperpiesis.

Practice tells us again that a permanently abnormal set of the arterial system does not take place until excessive stress has been in operation for a long time; how long we cannot say—probably the term depends in part on the resistance of the individual. But I think we may surmise that even after four or five years of hyperpiesis—if not extreme in degree—the heart and arteries, on condition of relaxation of pressures and unabated vigilance thereafter, may still be capable of recovery. I had written "prompt relaxation," but I deleted the adjective, for in these readjustments the more haste often the worse speed. *Festina lente*; the aberration has been attained gradually, and the process must be put back, if not as slowly, at any rate with due wariness, remembering that probably many readjustments are implicated. If there be a permanent set in a new form, as of leathery arteries and a thick heart, we shall have to act on a compromise, and be content with some result much less than the best. In the past history of not a few of my cases treated between 1870 and 1880 I found that by too determined an attempt to carry things back towards normal conditions I did more harm than good. The pressures were reduced indeed, but with them the patient's comfort and wellbeing also. In children, as we might anticipate, the reduction may be made pretty rapidly.

From these observations we pass easily to the rule that the high pressure is an attempt of the organism to maintain the equilibrium of its circulation. For some unknown reason the peripheral resistance is raised and must be overcome; if the machine is to go on. It is blind work to reduce the pressure without regard to these causes, unless some catastrophe, such as apoplexy, heart-failure, or angina pectoris be impending. Then the physician with his eyes open must intervene as the immediate conditions dictate. To meet such emergencies there are many harmless temporary expedients, such as a bleeding, a mercurial purge, and complete rest. If in spite of these, and such measures as these, the peril is not thrust back vaso-dilators may be—often must be—introduced, and their effect carefully watched, all demands upon the organism being warded off meanwhile.

The principles of the chronic treatment of the chronic malady, unknown as its causes are, happily rest upon a trusty, though an empirical, foundation. The subjects of hyperpiesis, if—as I have said frequently elsewhere—not usually themselves subject to gout, often come of gouty stock; the poisons, or parabolisms (if I may coin a word), though not identical, are probably akin. The general plan of treatment which answers for the one usually answers for the other—namely, strict order and moderation in diet, with exclusion of the purin bodies, abstinence or virtual abstinence from alcohol,* regular exercise, courses of deobstruent mineral waters, either at home or at a spa, "alterative" drugs, including the occasional use of mercury, and perhaps prolonged courses of iodide of potassium. This last drug I have conscientiously used for many years in hyperpiesis because I feel under some obligation to give my patients the chance of a benefit which is believed by good observers to be thus obtained. Vaso-dilators I use on the principles already set forth—that is, on emergencies only, and with some unwillingness; they do not touch the causes of a hyperpiesis which in intention is conservative, and in part they reduce it by depressing the heart. I generally begin treatment with a substantial dose of calomel, followed by small alterative doses of it—say, a quarter of a grain daily—for ten or twelve days; then I prescribe for a much longer time a small dose of podophyllin—such as a tenth of a grain or less thrice daily, or a quarter of a grain of iridin, with occasional but not infrequent returns to the mercury.

Sometimes, nay often, under such measures and precautions as these the hyperpiesis clears up quickly, and indeed more or less permanently; still it is wise in anyone who has observed in himself such a change to be watchful of himself thereafter. The condition may recur; but I have often noticed even then that if the distemper be chased away three or four times it may not recur. This result I have seen in not a few cases which have been under my occasional observation for many years. Perhaps it is that the hygienic precautions become habitual. There is one systematic cure or preservation in incipient or mildly recurrent cases which is better than spas and medicines, and this is mountain-climbing. Let the person thus liable get away twice a year into the hills, as to Switzerland, Cumberland, or Scotland; not to shoot driven game, to eat a hot lunch, and ride home in a motor-car, but to march up and down hill for a month from morn to eve, with no more than a crust of bread and a handful of prunes in his pocket. This I do in Switzerland and in the Lakes, keeping a watch on my blood pressure. I have never again had to go to Homburg, as I had to do some summers ago, when for two years I had been barred by other engagements from my climbing propensities. After a week or ten days on the glaciers the pulse will become as gentle as the pulse of a child.

When man had to hunt for his food, in obtaining foreign tissues he used up much of his own; moreover, pretty often he used himself without recompense. Nowadays food comes to the sedentary man and to the idle man regularly and abundantly, without bodily effort. Mental work does not consume the body as muscular work does—so much less that it is difficult to estimate a loss scarcely larger than the limits of experimental error; and yet, unfortunately, mental work engenders a hunger no less keen—keener, it would seem—than labour of the muscular frame. And much so-called exercise is indolence. Shooting especially has been tempered to the habits of the laziest gunner. Now all these people—the hot luncheon and brown sherry folk, as we may call them—are doing their best to send up their arterial pressures, and in most of these an ascetic rule will divert the march to the broad gate of arteriosclerosis, cardiac strain, angina pectoris, apoplexy, or acute pneumonia. The agricultural labourer, on the other hand, avoids hyperpietic arteriosclerosis to fall prematurely into the involutionary form. In towns the labourer is helped down hill by alcohol and syphilis.

But in some persons the proclivity to hyperpiesis is so strong as to defy ascetic rules, medicines, spas, and muscular drill. Then only by a patient adaptation of such means to the individual case can we make way, and too often the patient tires of such tedious preoccupations, slides back into his accustomed ways, and takes fright too late when one morning he has a hitch in his speech and a benumbed right hand, or finds that he cannot ascend his office stairs without stopping on the landing to catch his breath. Thenceforth there is no more than patching to be done. Still, with all obedience, it is impossible in some of these patients to reduce the pressure, or at any rate to kept it down; frequently, indeed, the state of things is long overlooked, because the radial artery is so closely contracted that to the unsuspicious finger the high pressure is not apparent; in an artery so narrow, and impinging on so small an area of the finger, a systolic pressure of over two hundred may hardly be perceived without the sphygmometer. It would seem as if the vaso-motor centre were held up by some inexorable poison. By their hypertonic resistance the arteries save their coats, but the comparatively atonic vessels of the brain suffer the more, and these cases usually terminate in an apoplexy.

* I do not find that alcohol causes atheroma, but it is a potent ally of more specific causes. Lead, for example, acts far more rapidly in those who consume alcohol freely.

